# Glucose transport in the regulation of T-cell activation: the journey may be as important as the destination

**DOI:** 10.1097/IN9.0000000000000003

**Published:** 2022-08-05

**Authors:** Steven W. Barger

**Affiliations:** 1 Department of Geriatrics, University of Arkansas for Medical Sciences, Little Rock, AR, USA; 2 Department of Neurobiology & Developmental Sciences, University of Arkansas for Medical Sciences, Little Rock, AR, USA; 3 Geriatric Research, Education & Clinical Center, Central Arkansas Veterans Healthcare System, Little Rock, AR, USA

**Keywords:** gene expression, glucose, glucose transporter, metabolism, T cells

## Abstract

A shift in the energy-metabolism balance from oxidative phosphorylation to glycolysis is observed in several phenomena, from oncogenesis to differentiation. And this shift is not merely an indicator of the phenotypic change—an increase in glucose delivery often drives the adaption. At first blush, it seems that any route of entry should be equivalent, as long as sufficient quantities are supplied. However, an extensive study comparing the Th17 and Th1 subtypes of T cells now suggests that similar glucose transporters may not be interchangeable. Manipulation of individual transporters, or the downstream metabolites of their substrates, may afford dampening of autoimmunity potential with some degree of precision.

## Graphical abstract

**Figure:**
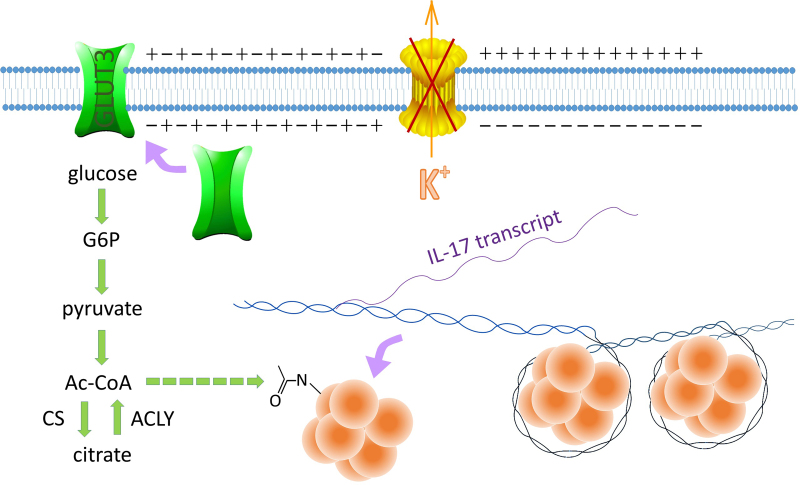

Differential effects have been described for simple, diffusible molecules depending on localization in microdomains, route of access to those microdomains, or the kinetics of their change. Such differences often appear to be possible due to binding partners or enzymes that have exquisitely distinct affinities for the ion or molecule in question. For instance, calcineurin localized near lysosomes has a very high affinity for calcium and therefore can be activated by small releases of the ion from this organelle to activate a transcription factor that critically impacts lysosomal function ^[1]^. Affinities distinguish several of the facilitative glucose transporters, but two of the most extensively studied, GLUT1 and GLUT3, have very similar affinities for their substrate (although this depends on methodologies of measurement and interpretations thereof ^[2]^). Their different roles are often attributed to their expression in different cell types; GLUT3 is typically thought of as the “neuronal” glucose transporter. Nevertheless, alterations in the metabolism of a fuel as simple and universal as glucose can have very different effects in different cell types or life stages. This is perhaps most evident in the oncogenic transformation of tumor cells, which nearly always involves an increase in ATP production from glycolysis at the expense of using that glucose to ultimately contribute a carbon source to oxidative phosphorylation (OXPHOS) in mitochondria—a shift known as the Warburg Effect ^[3]^. This is understandably adaptive, as tumors both tend to outgrow a sufficient oxygen supply and, maybe more importantly, develop a ravenous need for amino acids such as serine and glycine, which can be generated as byproducts of glycolysis ^[4]^. Perhaps for similar reasons, activation of immune cells—particularly, in the direction of inflammatory sequelae—likewise involves something like a Warburg shift, even when oxygen is abundant. Macrophages that differentiate into a proinflammatory state show higher rates of glycolysis than those that have a more reparative, trophic-support phenotype ^[5]^. And this shift is not merely permissive for aggressive activity; it can actually drive differentiation in a particular path, as evinced by the production of higher levels of inflammatory cytokines in macrophages forced to overexpress GLUT1 ^[6]^.

Metabolic changes analogous to those occurring in macrophages also prevail in the activation T cells ^[7]^. Whereas naïve T cells rely largely on OXPHOS, including considerable β-oxidation of lipids, memory T cells gobble up much more glucose, applying it to very efficient ATP production through aerobic glycolysis (that occurring when oxygen is not limiting) and even finding the opportunity to sock some of it away through the synthesis of fatty acids. Blocking this glycolytic shift acts as a checkpoint, preventing activation. A wide variety of models and outcome measures have extensively documented a role for GLUT1 in T-cell maturation ^[7]^, although subtleties have been noted. For instance, CD8^+^ T cells preferentially elevate protein levels of GLUT3 over GLUT1 under specific conditioning ^[8]^.

Hochrein et al have now reported an extremely thorough and extensive study documenting an essential role for GLUT3-mediated elevations of glucose, along with key roles for its downstream metabolites, in the maturation and function of Th17 cells ^[9]^. Of course, Th17 cells play critical roles in several conditions that are in some fashion autoimmune. A very keen interest has developed in this class in multiple sclerosis, as well as its primary model experimental autoimmune encephalitis (EAE). Through a juggernaut of experiments with ex vivo endpoints and in vivo confirmations, they show that restriction of GLUT3 expression through conditional knockout (cKO) in CD4^+^ T cells alters Th17 phenotype in remarkable ways, and overexpression pushes several of these endpoints in the opposite direction.

Importantly, the team found that ATP levels were not affected in GLUT3-cKO CD4^+^ cells, though proton efflux rate (PER; essentially, lactate production) was diminished. GLUT3-cKO Th17 cells also showed slightly enhanced apoptosis and thus diminished growth rates. However, mitosis did not appear to be affected, distinguishing this transporter’s effects from those reported for GLUT1 ^[10]^. Also unaffected was IFNγ production. But, IL-17, IL-2, and GM-CSF were generally reduced greatly. Moving on to disease models, Hochrein et al found that EAE was dramatically attenuated in GLUT3-cKO mice, both confirming the critical role for Th17 cells in this paradigm and pointing up the translational potential of using GLUT3 (or its downstream products) therapeutically. Caution is warranted, of course, as it seems every aspect of immune modulation can have Janus-faced outcomes; bacterial infection (*Citrobacter rodentium*) was worsened in the GLUT3-cKO mice.

GLUT3 modulation made a large impact on gene expression; in Th17 cells, differentially expressed genes (DEG) trended toward favoring reductions in expression level. Somewhat unexpectedly, cKO mice even had reduced expression of some of the genes involved in mitochondrial respiration (so they did not compensate for the reduced glycolysis through boosted OXPHOS). Another interesting aspect is the degree to which differences were noted between Th1 and Th17 cells regarding the impact of GLUT3-cKO on genes (there were only 217 shared DEG out of 460 and 933 DEG in the respective cell types) and on TCA cycle intermediates. Intermediates derived from [^13^C]glucose were assessed; consistent with compromised OXPHOS, those in the TCA cycle—fumarate, malate, and acetyl-CoA—were significantly decreased in GLUT3-cKO Th17 cells.

It might surprise some that glucose metabolism should have such a profound effect on gene expression. However, there is a fascinating relationship that places the staples of energy metabolism in direct modulation of gene expression: ATP-citrate lyase (ACLY) is capable of converting citrate back into acetyl-CoA (and oxaloacetate). This occurs with some abundance in the nucleus, thereby shuttling the acetyl-CoA product into histone modification. Acetyl-CoA is the substrate for histone acetyltransferases (HATs), which play crucial roles in transcriptional regulation. Transfer of an acetyl group from acetyl-CoA to a histone’s lysine residues promotes release of DNA from the nucleosome, a critical step in opening up the structure for access by the transcriptosome ^[11]^. Thus, aerobic glycolysis need not produce excessive lactate; the conversion of glycolytic pyruvate into acetyl-CoA, and even citrate, can still occur without feeding the TCA cycle. Notably, HATs with low affinity for acetyl-CoA are more sensitive to acetyl-CoA depletions; the acetylation of Lys9 on H3 histone (H3K9) appears to be among the most tenuous ^[12]^. Hochrein et al evaluated the role of this site in their model via both genetic and pharmacological inhibition of ACLY. Each of these approaches attenuated IL-17 production, and the pharmacological inhibitor (2-hydroxycitrate) ameliorated autoimmunity in EAE. H3K9 and the genes it controls seemed to be particularly important, including IL-17 itself.

As intriguing and clinically promising as this study is, it also leaves some important questions to be answered. Why, for instance, would the GLUT3 elevation in activated Th17 cells be associated with a lactate boost if the carbon sources it provides were primarily destined for acetyl-CoA and becoming chronically trapped in histone acetylation? Presumably, the latter can be accomplished with a small portion of the total glucose; but if so little is required, how does GLUT3-transported glucose become rate-limiting for gene regulation?

Regardless, the most intriguing issue may be the specificity of a requirement for GLUT3 when metabolic transformation of so many other cell types—including most T cells—occurs just fine with GLUT1. It seems somewhat surprising that inhibition of ACLY by the Würzburg team had effects that were relatively specific for IL-17 production in mouse T cells. If off-target effects are eventually discovered, exploiting the various requirements of T-cell subtypes for distinct transporters might afford greater therapeutic specificity. As mentioned above, the specific roles of GLUT1 and GLUT3 would not seem to depend on their relative affinities for glucose, which are quite similar. One clue may lie in Figure S1A,B, showing a dramatic predominance of the GLUT3 localization to the plasma membrane. In endothelial cells, adipocytes, and most other cell types examined, a strong majority of GLUT1 is distributed to the plasma membrane too. But, this need not be the case. We find that astrocytes, which express a modified version of GLUT1, typically traffic less than half of this transporter to the cell surface; even less so under inflammatory conditions ^[13]^. The characterization of GLUT3 as the “neuronal” glucose transporter may not be irrelevant here. Depolarization of a neuron’s excitable membrane drives insertion of GLUT3 into a functional position in the lipid bilayer ^[14]^, and T-cell activation can alter membrane potential, suppressing IL-2 release, and some other phenotypic responses ^[15]^. Perhaps, this study points to something about T-cell biology that is “exciting” in more ways than one!

## Conflicts of interest

The author declares that he has no conflicts of interest.

## Funding

Dr. Barger receives funding from NIH/NIA (1R01AG071782).
